# Pulmonary Tuberculosis

**Published:** 1918-12

**Authors:** 


### PULMONARY TUBERCULOSIS

With the present grippe and pneumonia epidemic and with the coming of winter, the three collecting centres in the A. E. F. for the sorting out of the pulmonary cases will be greatly taxed.

Most of the American Divisions were weeded of manifest pulmonary tuberculosis before coming to France. Possibly 7/10 of 1 o/o of the men were discarded by special tuberculosis examiners after they had passed through medical examinations into the Federal service.

Without repeated weeding a garden is soon overrun, and so in the recognition and evacuation of cases of pulmonary tuberculosis medical officers must be constantly alert.

Conditions regarding this disease in the French Army and among the civilians are far less serious than at first reported. Instead of our army, except in cities of the S. O. S., being in danger of infection by contact with the French people, possibly French children in communes and villages in the parts of France hitherto almost free of tubercle, may be exposed to danger from our, so far, rare cases which are not promptly evacuated.

At present many slowly convalescing cases of grippe and pneumonia are being mistaken for pulmonary tuberculosis both by clinicians and roentgenologists. Careful observation over several weeks, repeated examinations, and the results of the employment of drainage by posture are all necessary in deciding the disposition of these men when sputum is not positive to tubercle.

Autopsies in general, especially at Evacuation Hospitals, show a negligible number of latent lesions,

If we can judge American manhood at twenty-five years by British manhood of similar age it may be found that pulmonary tuberculosis causing death is rapidly disappearing at this life period.

By means of original methods of dissecting statistics, Brownlee in England has indicated that there are probably different types (as with dysentery, typhoïd, meningococcus, pneumococcus, tetanus) of the human tubercle bacillus.

Type I causes deaths at 20 to 25 years of age.
Type II causes deaths at 45 to 50 years of age.
Type III causes deaths at 55 to 65 years of age.

Now in most sections of the British Isles deaths from Type I have very greatly diminished in recent years; the number is only half that of the deaths occurring from Type II at 45 to 50 years. The autopsy observations above referred to would seem to agree with these expectations, therefore, and the problem of having large numbers of tuberculous soldiers to deal with is not anticipated.

Inasmuch as the tuberculous "wounded" receive no honors, are liable to spend much time at "sick call", can endanger their fellow soldiers through massive doses of tubercle bacilli, can also infect French villages, it is from every point of view necessary to have them quickly evacuated to the excellent Army Sanatoria which the government has supplied at home. This can only be brought about by frequent examinations of men who get short of breath, spit more than a mouthful of blood, who tire easily, or who rapidly are losing weight.

The fact that the disease is rare rather than common in the A. E. F. should be the greater incentive to its recognition.

